# Supplementary material for: Recapitulation of HDV infection in a fully permissive hepatoma cell line allows efficient drug evaluation
Source: Nat Commun. 2019 May 22;10:2265. doi: 10.1038/s41467-019-10211-2 (PMC6531471; doi:10.1038/s41467-019-10211-2)
Supplement: Supplementary file 1 — Supplementary Information [file 41467_2019_10211_MOESM1_ESM.pdf]

SUPPLEMENTARY INFORMATION

Recapitulation of HDV infection in a fully permissive hepatoma cell line allows efficient drug evaluation

Florian A. Lempp[1,2], Franziska Schlund[1], Lisa Rieble[1], Lea Nussbaum[1], Corinna Link[1], Zhenfeng Zhang[1], Yi Ni[1,2] & Stephan Urban[1,2,*]

[1] Department of Infectious Diseases, Molecular Virology, University Hospital Heidelberg, Heidelberg, Germany;

[2] German Centre for Infection Research (DZIF), partner site Heidelberg, Heidelberg, Germany

* Correspondence: stephan.urban@med.uni-heidelberg.de

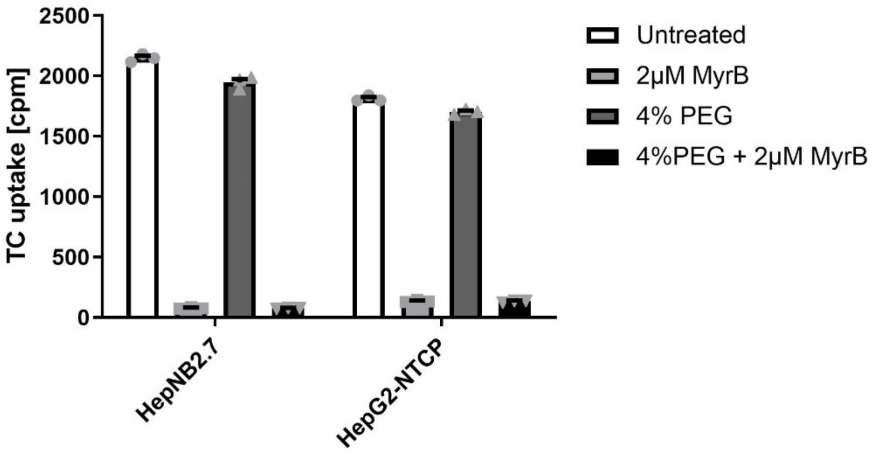

Supplementary Figure 1: Incubation with PEG does not influence NTCP transporter function in HepNB2.7 cells. HepNB2.7 or HepG2-NTCP cells were seeded in 24-well plates and incubated with [3H]-TC in the presence or absence of 2 µM MyrB and/or 4% PEG. Cells were washed extensively and intracellular [3H]-TC was quantified by liquid scintillation counting.

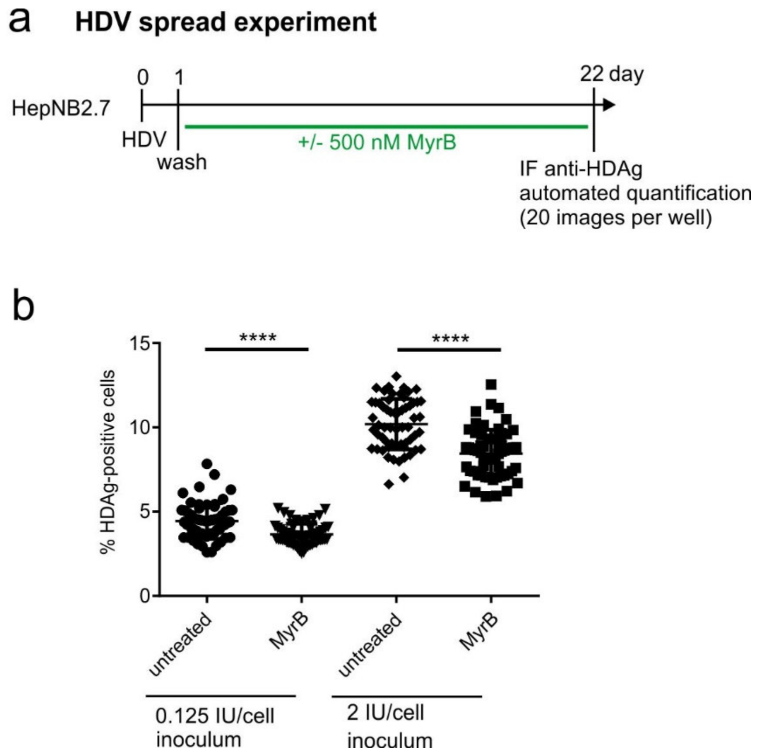

Supplementary Figure 2: HDV spread is limited in HepNB2.7 cells. (a) Schematic representation of the experimental layout: HepNB2.7 cells seeded in 24-well plates were inoculated with 0.125 or 2 IU/cell HDV. One day post infection, the inoculum was removed and fresh medium with or without 500 nM MyrB was added and replaced every 2nd/3rd day thereafter until day 22. Cells were fixed, HDAg was immunostained and positive cells were quantified by automated image acquisition and analysis (b). 20 images per well were analysed. Data shows the mean of three biological replicates. Unpaired two-tailed Student's t-test was used for statistical analyses (*P<0.05, **P<0.01, ***P<0.001, ****P<0.0001, n.s. not significant P>0.05).

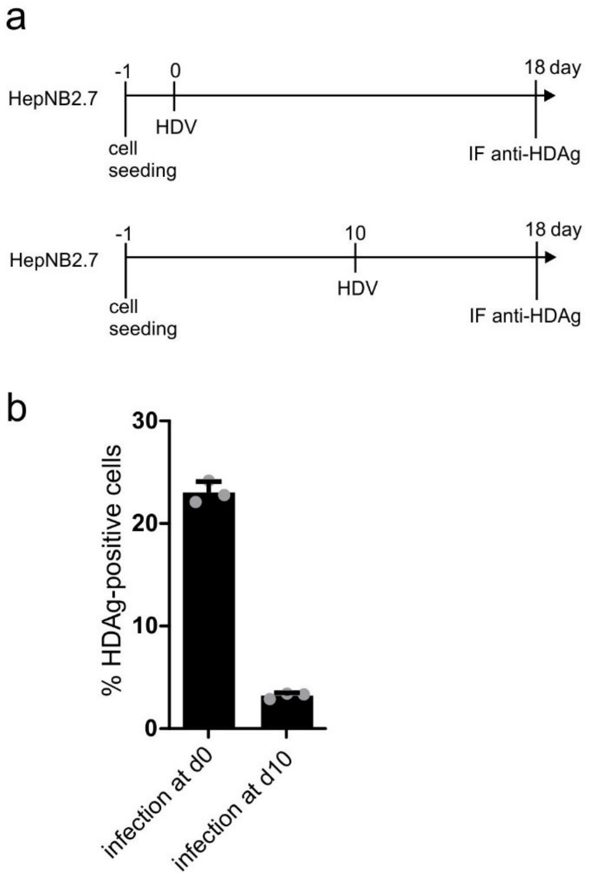

Supplementary Figure 3: HepNB2.7 cells lose susceptibility to HDV after long-term culture. (a) Schematic representation of the experimental layout: HepNB2.7 cells were seeded in 24-well plates and inoculated with HDV the next day (d0) or eleven days later (d10). At d18, cells were fixed and immunostained for HDAg. (b) HDAg-positive cells were quantified.

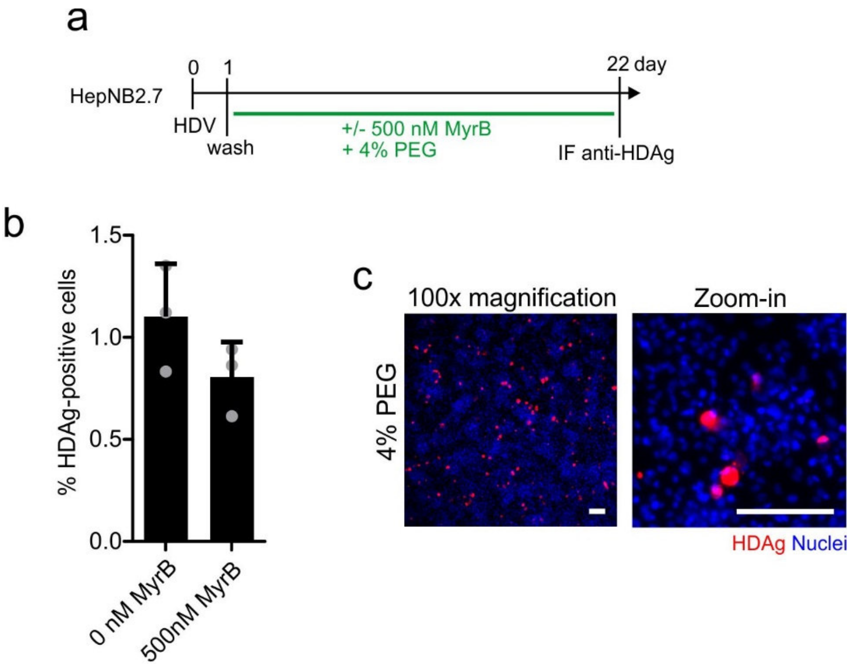

Supplementary Figure 4: Continuous PEG treatment does not induce viral spread in HepNB2.7 cells. (a) Schematic representation of the experimental layout: HepNB2.7 cells seeded in 24-well plates were inoculated with 0.125 IU/cell HDV. One day post infection, the inoculum was removed and fresh medium containing 4% PEG with or without 500 nM MyrB was added and replaced every 2nd/3rd day thereafter until day 22. Cells were fixed, HDAg was immunostained and positive cells were quantified by automated image acquisition and analysis (b). 1 image per well were analysed. Data shows the mean of three biological replicates. (c) A representative immunofluorescence image of cells continuously treated with 4% PEG for a total of 21 days is shown (scale bars: 100 µm).

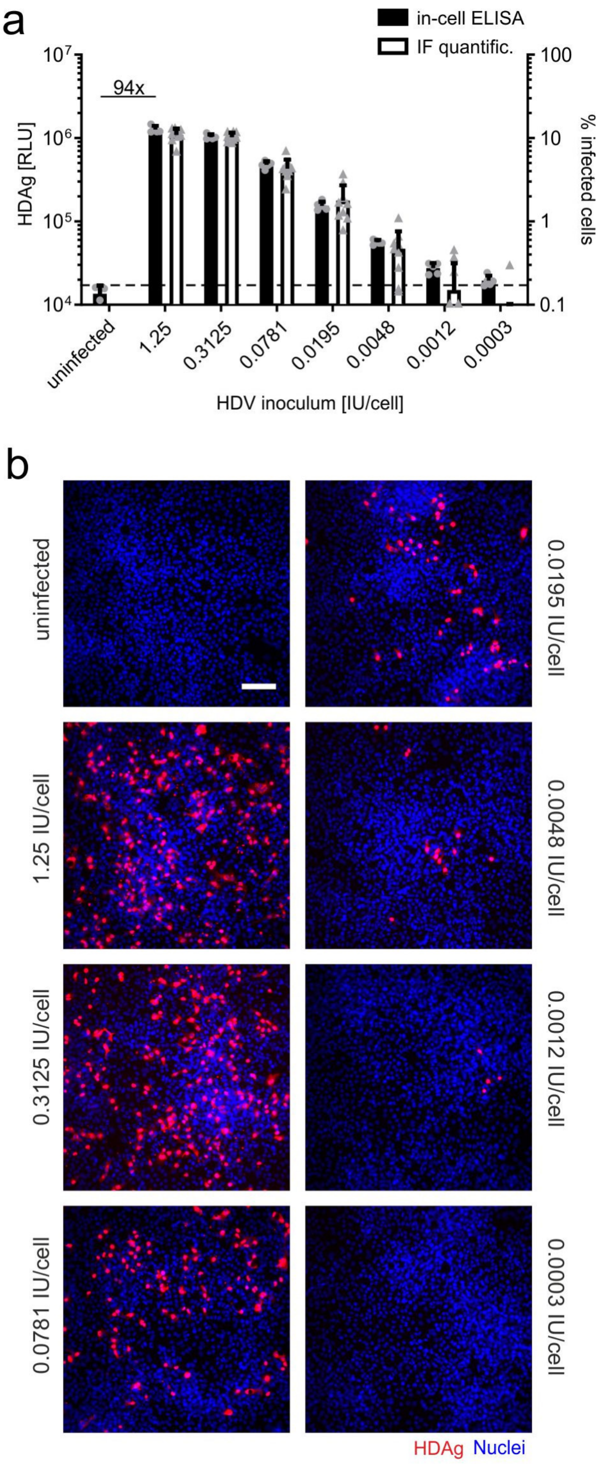

Supplementary Figure 5: Quantification of HDV infection by in-cell ELISA in HuH7-NTCP cells. HuH7-NTCP cells seeded in a 96-well plate were inoculated with 4-fold dilutions of HDV starting from 1.25 IU/cell. Cells were fixed at day 8 post infection and (a, black bars) in-cell ELISA was performed or (b, scale bar: 100 µm) immunofluorescence staining of HDAg was performed and positive cells were quantified by automated image analysis (a, white bars).

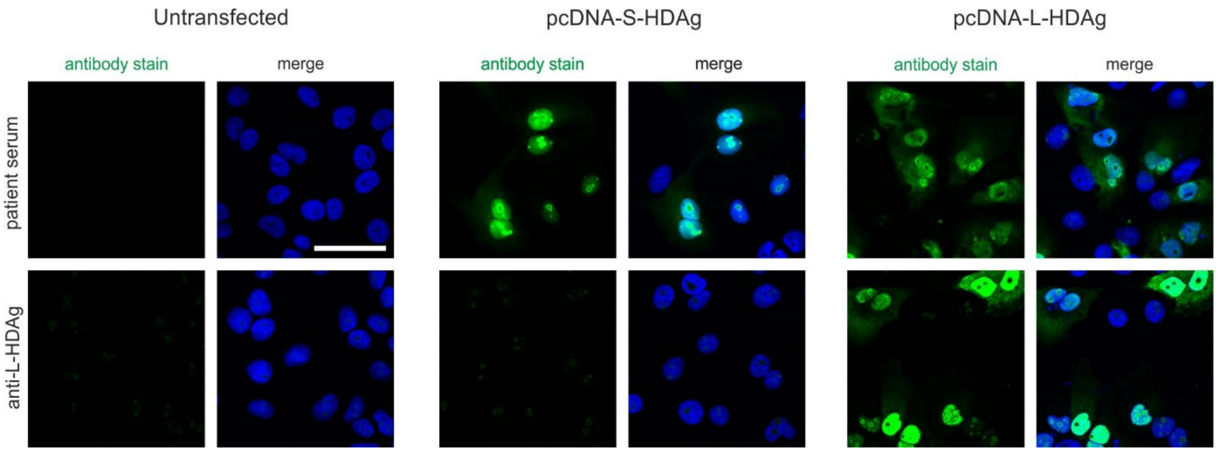

Supplementary Figure 6: Specificity of the L-HDAg-specific polyclonal antibody. HuH7 cells were transfected with a plasmid encoding S-HDAg (middle), L-HDAg (right) or remained untransfected (left). Two days post transfection, cells were fixed and immunostained with a patient serum containing antibodies against total HDAg (upper) or with a polyclonal rabbit serum containing antibodies specific for L-HDAg (lower). Alexa488-coupled secondary antibodies were used for fluorescent labeling. Images were obtained on a confocal microscope (Leica sp8, scale bar: 50 µm).

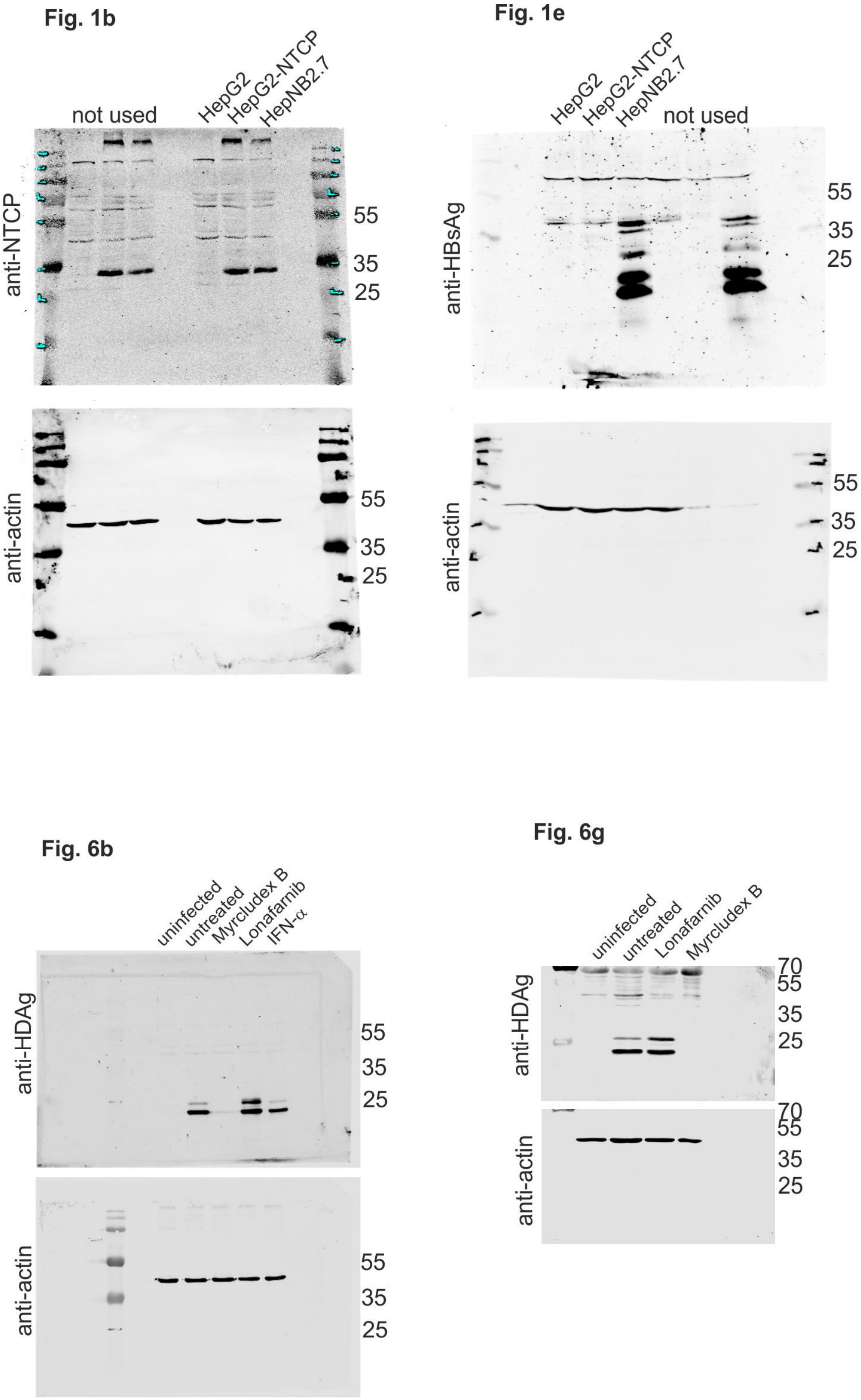

Supplementary Figure 7: Uncut images of all Western Blots. Subpanels are labeled according to their appearance in main figures. Protein molecular weight markers are annotated in kDa.

Supplementary Table 1: List of all primers used in this study

Primer name	Sequence	Reference
HB27-Sma-f	CAGTCCCGGGCAATCTCGGGAATCTCAATG	
HB27-Nhe-rev	CAGTGCTAGCCTCGTACTGAAGGAAAGAAG	
Ferns-HDV-f	GCGCCGGCYGGGCAAC	[1]
Ferns-HDV-r	TTCCTCTTCGGGTCGGCATG	[1]
Ferns-HDV-probe	5'FAM-CGCGGTCCGACCTGGGCATCCG-3'TAM	[1]
IFIT1-f	AGAAGCAGGCAATCACAGAAAA	
IFIT1-r	CTGAAACCGACCATAGTGGAAAT	
IFNb-f	ACCAACAAGTGTCTCCTCCA	[2]
IFNb-r	AAGCCTCCCATTCAATTGCC	[2]
IFNl1-f	CGCCTTGGAAGAGTCACTCA	[2]
IFNl1-r	GAAGCCTCAGGTCCCAATTC	[2]
RSAD2-f	CGTGAGCATCGTGAGCAATG	[2]
RSAD2-r	TCTTCTTTCCTTGGCCACGG	[2]
Mx1-f	AAGAGCCGGCTGTGGATATG	[2]
Mx1-r	GGCGGTTCTGTGGAGGTTAA	[2]

Supplementary references:

1 Ferns, R. B., Nastouli, E. & Garson, J. A. Quantitation of hepatitis delta virus using a single-step internally controlled real-time RT-qPCR and a full-length genomic RNA calibration standard. *J Virol Methods* **179**, 189-194, doi:10.1016/j.jviromet.2011.11.001 (2012).

2 Zhang, Z. *et al.* Hepatitis D virus replication is sensed by MDA5 and induces IFN-beta/lambda responses in hepatocytes. *J Hepatol* **69**, 25-35, doi:10.1016/j.jhep.2018.02.021 (2018).
